# Economic Inequality Predicts Biodiversity Loss

**DOI:** 10.1371/journal.pone.0000444

**Published:** 2007-05-16

**Authors:** Gregory M. Mikkelson, Andrew Gonzalez, Garry D. Peterson

**Affiliations:** 1 McGill School of Environment, Montréal, Québec, Canada; 2 Department of Philosophy, McGill University, Montréal, Québec, Canada; 3 Department of Biology, McGill University, Montréal, Québec, Canada; 4 Department of Geography, McGill University, Montréal, Québec, Canada; CNRS, France

## Abstract

Human activity is causing high rates of biodiversity loss. Yet, surprisingly little is known about the extent to which socioeconomic factors exacerbate or ameliorate our impacts on biological diversity. One such factor, economic inequality, has been shown to affect public health, and has been linked to environmental problems in general. We tested how strongly economic inequality is related to biodiversity loss in particular. We found that among countries, and among US states, the number of species that are threatened or declining increases substantially with the Gini ratio of income inequality. At both levels of analysis, the connection between income inequality and biodiversity loss persists after controlling for biophysical conditions, human population size, and per capita GDP or income. Future research should explore potential mechanisms behind this equality-biodiversity relationship. Our results suggest that economic reforms would go hand in hand with, if not serving as a prerequisite for, effective conservation.

## Introduction

Human activities have dramatically increased the rates of species and population extinction [1]. This directly undermines the richness and diversity of life on Earth [2], [3], and indirectly threatens human welfare, e.g., through negative effects of species loss on ecosystem services [4], [5]. The proximate causes of biodiversity loss are relatively well understood, with habitat destruction, climate change, biotic homogenization, resource extraction, and pollution the major factors [6], [7]. However, the socioeconomic forces behind these biophysical drivers are poorly known [8].

While the sheer size of a country's economy predicts its overall environmental impact reasonably well [9], little is known about how the distribution of wealth or income within an economy affects the environment. Olson [10] suggested that small groups with considerable inequality might favor the provision of a public good. The idea is that when the majority of the wealth is held by a few resource-users, it is in their interest to conserve regardless of what the poorer members of the group do. Some more recent theoretical analyses also support this perspective [11], [12]. However, others suggest that inequality may hinder conservation [13], [14], and empirical work has shown that inequality can thwart the collective action required for environmental protection [15] and public health [16]. Although these studies suggest a connection between inequality and environmental degradation, the sign and strength of the relationship with biodiversity remains unknown.

We therefore used new high-quality data to test whether and how strongly inequality is linked to biodiversity loss. We examined two different spatial scales – entire countries, and states within the US – and used the Gini ratio of income inequality as our measure of economic inequality. This statistic, applied to households at the country scale and families at the state scale, can theoretically vary between 0 and 1. 0 would indicate that all of the households or families in a given society have exactly the same income, while 1 would mean that a single household or family earns all of the income, with no one else receiving any. Actual Gini ratios have ranged from 0.16 to 0.68 among different countries and years between 1960 and 1999 [17], and from 0.31 to 0.53 among different US states and years between 1969 and 1999 [18].

Our measure of biodiversity loss in countries is the number of plant and vertebrate species known to be threatened in 2004 [19]. We implicitly controlled for biophysical variables, such as area and climate, by including a variable that is highly correlated with them, namely the total number of plant and vertebrate species (again, in 2004). We explicitly controlled for two socioeconomic variables, human population size [20] and gross domestic product purchasing power parity (GDP PPP) per capita. GDP PPP is an adjusted version of the GDP, ensuring that each dollar “buys an equivalent amount of goods or services irrespective of the country.” [21] We also included the square of GDP PPP per capita in our analysis, to permit detection of the non-linear “environmental Kuznets” relationships that some have proposed for environmental impacts – first increasing, but then decreasing, with per capita GDP [22].

Finally, we allowed for a time lag between socioeconomic causes and biological effects, rather than using contemporary data for all variables. We chose 1989 for our socioeconomic data, since that is the year for which Gini ratios are available for the largest number of countries: 61 [17]. Missing information about variables other than inequality limited our final sample size to 45 countries. Together these countries cover 51% of the Earth's land surface excluding Antarctica, and currently contain 62% of the world population and generate 71% of the gross world product [20], [21].

As an indicator of biodiversity loss within US states, we used the number of permanent resident bird species with statistically significant declines in abundance (*P*-value<0.10) over the period covered in the breeding bird survey, 1966–2005 [23]. Permanent residents are presumably the species most affected by within-state socioeconomic conditions. We also controlled for the total number of permanent resident bird species in 2005, and human population size [24] and per capita income [25]. Again allowing for a time lag between socioeconomic causes and biological effects, our state inequality and per capita income data are for 1969, and population data for 1970 – the years for which these socioeconomic statistics are available and that are closest to the start of the bird monitoring period in 1966. For five states, our sources lack information about one or more of the variables in our analysis. So our sample size at this scale is also 45, with these 45 states collectively extending over 91% of the US land surface, containing 97% of its human population, and accounting for 97% of its total income [24], [25].

We used multiple regression to analyze the data described above. Analysis of residuals warranted the use of a power model at the country scale. This accords with previous studies finding power relationships between countries' biophysical and socioeconomic characteristics and their environmental impacts [9], [26]. For US states, residual analysis warranted a linear model. See Materials and methods for more detail.

## Results

**Figure 1:**
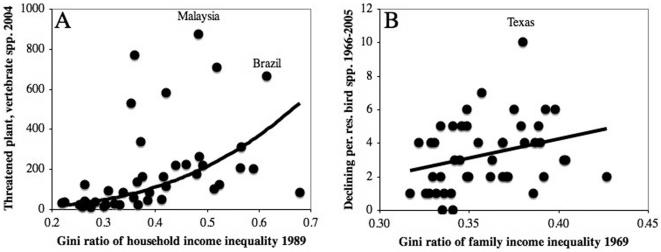
Relationships between the Gini ratio of income inequality and early indicators of biodiversity loss. (A) Number of threatened plant and vertebrate species across countries; the curve shows the best-fit bi-variate power relationship. (B) Number of declining permanent resident bird species across US states; the line shows the best-fit bi-variate linear relationship. Of the apparent outliers in both Figure 1A and 1B, only those identified in the course of the multi-variate analyses described in the text are labeled.

Among both countries and states, we found striking relationships between income inequality and biodiversity loss. As Figure 1 shows, societies with more unequal distributions of income experience greater losses of biodiversity. After other variables have been taken into account, the country-level Gini ratio of household income inequality in 1989 has a highly significant power relationship with the number of threatened plant and vertebrate species in 2004 (*P* = 6.4×10^−6^). The estimated inequality exponent is 1.76, which means that a 1% increase in the Gini ratio is associated with an almost 2% rise in the number of threatened species. Inequality is even more significant (*P* = 1.1×10^−6^) after removing statistical outliers (Brazil, Jamaica, Kyrgyzstan, Malaysia, and New Zealand). Alternative models confirm this link between economic inequality and biodiversity loss (see Table 1 and Materials and methods).

**Table 1:** Parameter estimates and performance statistics of models predicting biodiversity loss.

*Countries*								
	Parameter estimates (standard errors)					Performance statistics		
Model	Total number of plant and vertebrate species 2004	Human population size 1989	GDP PPP per capita 1989	GDP PPP per capita 1989 (quadratic)	Gini ratio of household income inequality 1989	*P*-value of inequality	*ΔAIC_C_*	*r* ^2^
Power	**0.81** (0.16)	0.10 (0.08)	−0.03 (1.58)	2.8×10^−3^ (0.09)	**1.76** (0.34)	6.4×10^−6^	21.0	0.86
Linear	**0.01** (2.3×10^−3^)	**2.6×10^−7^** (1.2×10^−7^)	−2.1×10^−3 ^(.01)	1.5×10^−7 ^(6.6×10^−7^)	**445.7** (208.8)	0.04	2.2	0.74
Negative binomial	**6.1×10^−5^** (1.1×10^−5^)	9.7×10^−10^ (6.1×10^−10^)	2.9×10^−5 ^(7.2×10^−5^)	−1.2×10^−9^ (3.3×10^−9^)	**5.80** (1.05)	3.0×10^−8^	16.7	–

*US states*								
	Parameter estimates (standard errors)					Performance statistics		
Model	Total number of permanent resident bird species 2005	Human population size 1970	Per capita income 1969	Per capita income 1969 (quadratic)	Gini ratio of family income inequality 1969	*P*-value of inequality	*ΔAIC_C_*	*r* ^2^
Linear	**0.09** (0.04)	1.5×10^−7^ (7.4×10^−8^)	2.9×10^−3^ (1.8×10^−3^)	−1.2×10^−7^ (7.4×10^−8^)	**33.6** (13.9)	0.02	3.5	0.53
Power	0.28 (0.21)	**0.24** (0.08)	59.8 (41.4)	−3.19 (2.22)	**2.76** (1.30)	0.04	2.1	0.52
Negative binomial	0.02 (0.02)	4.0×10^−8^ (2.6×10^−8^)	1.1×10^−3^ (6.4×10^−4^)	−4.5×10^−8^ (2.8×10^−8^)	**11.1** (4.9)	0.02	2.2	–

The dependent variable is the number of threatened plant and vertebrate species in 2004 (for countries) or of permanent resident bird species with statistically significant (*P*<0.10) declines in abundance from 1966 to 2005 (for US states). The sample size is 45 for both countries and states. For the power models, all variables were log-transformed before performing the regressions. Prior to log-transforming the numbers of declining permanent resident bird species in US states, however, we added 1 to each, since some US states had no declining species, and the logarithm of zero is undefined. For the linear and negative binomial models, no variables were log-transformed. See the Materials and methods section for more details, including an explanation of the quadratic term of GDP PPP/income per capita. Statistically significant parameter estimates are in bold (*P*<0.05). *ΔAIC_C_* is the advantage, in terms of Akaike's Information Criterion corrected for small sample sizes, of the model shown, in comparison to the corresponding null model with the inequality term removed [27].

Among US states, the Gini ratio of family income inequality in 1969 has a significant linear relationship, after controlling for other variables, with the number of permanent resident bird species that experienced significant declines in abundance between 1966 and 2005 (*P* = 0.02 for inequality). Once again, this result is robust to the exclusion of outliers (California, Maryland, New York, South Carolina, Texas, and Washington; *P*-value of inequality = 4.0×10^−3^), and confirmed by alternative models. Among both countries and states, inequality remains significant if the percentage of extant species that are threatened or declining, rather than the raw number of threatened or declining species, is used as the dependent variable (*P* = 6.4×10^−6^ and 0.02, respectively).

We tested the appropriateness of our socioeconomic variables in two ways. First, we tried all possible time lags for which our data allow a sample size of at least 20. The results support our original choices of time lag, and indicate how the strength of the relationship between economic inequality and biodiversity loss varies across different time lags. For most time lags, this relationship is stronger than those found between biodiversity loss and either human population size or affluence. See Table S1 in the supplementary information for more information. Second, we checked how well changes over time in socioeconomic variables, rather than values at a single time, explain biodiversity loss. Except for the change in per capita GDP at the country level, such changes do not correlate significantly with threatened or declining species (*P*-value of change in inequality = 0.16 for countries and 0.98 for US states).

Finally, we did one more check on the robustness of our results at the country scale, and one more on the appropriateness of our dependent variable at the US state scale. For countries, we tested whether inequality remains significant after controlling for geography, and for the demise of communist regimes. Dummy variables were used to indicate whether a country is in Africa, Asia, Australasia, Europe, or Latin America; and whether it is ex-communist or not. (An additional dummy variable for North America was not required, since only one country in our analysis – the US – is in that continent.) In a power model with the biophysical and socioeconomic variables used in the main analysis, plus the five geographic and one historical dummy variable just mentioned, the Gini ratio in 1989 still has a statistically significant, positive relationship with the number of threatened species in 2004 (*P* = 0.03).

For states, we tested whether a measure of biodiversity gain, rather than loss, has any relationship with economic inequality. (No comparable statistics on biodiversity gain are available at the country scale.) After controlling for other variables, the Gini ratio in 1969 has a negative linear relationship with the number of permanent resident bird species that experienced significantly positive trends in abundance 1966–2005. Although this negative relationship is not statistically significant (*P* = 0.19), it rules out the possibility that unequal states might simply have greater species turnover than more equal states. If inequality only increased gross turnover, rather than net biodiversity loss, then both declining and increasing species would be positively correlated with inequality.

## Discussion

We have thus demonstrated a striking correlation between economic inequality and biodiversity loss. While our findings cohere with previous work showing links between inequality and human health [16], they contrast with previous research suggesting that the overall size of an economy (i.e., population times per capita GDP or income) is the primary driver of environmental impacts [9], [26]. According to one cross-country analysis of per capita GDP and threatened species, the numbers of threatened species in most taxa follow a U-shaped pattern: first falling, but then rising, with increasing per capita GDP [26]. This is the opposite of the hump-shaped “environmental Kuznets” relationship expected by many economists between affluence and its environmental impacts. We used very similar data on threatened and total species; and we also allowed for detection of monotonic, U- shaped, and hump-shaped relationships; by adding a quadratic term for GDP PPP per capita. Nevertheless, we did not find any such patterns. This may be partly due to sample size (45 countries in our analysis, as opposed to more than 100 [26]). But the previous study also did not include inequality, or allow for a time lag between socioeconomic causes and biological effects, as we have.

Future research could test the generality of the link between economic equality and biological diversity, e.g., by examining states or provinces in countries other than the US. Further studies are also needed to establish the degree to which this link arises from common influences on both variables vs. direct effects of equality on biodiversity. In this analysis, we took two steps toward proving a direct causal relationship. First, we controlled for several likely common causes, and second, we incorporated time lags that are more realistic than any instantaneous effect of equality on biodiversity would be. Controlling for other potentially confounding variables – e.g., the degree to which different societies are governed democratically – could further test the extent to which this relationship is causal. But perhaps most importantly, future studies should explore possible mechanisms.

If such research confirms a causal relationship, it may help to predict future impacts of the rising inequality that most countries, as well as US states, have suffered over recent decades [17], [18]. For example, given that the Gini ratio in the US rose by 5% from 1989 to 1997, the country-level power model described in Table 1 suggests that we should expect a roughly 9% increase in the number of threatened plant and vertebrate species there by 2012. And we might expect the 3% rise in British inequality from 1989 to 1996 to result in a 5% increase in threatened species there by 2011. In general, unless current trends toward greater inequality are reversed, it may become increasingly hard to conserve the rich variety of the living world. Conversely, if we can learn to share economic resources more fairly with fellow members of our own species, it may help us to share ecological resources more fairly with our fellow species.

## Materials and Methods

All statistical analyses were performed with the software package R (Version 2.4.1), freely available at www.r-project.org. For linear models, we performed ordinary least squares regressions (the “lm” command in R) of the number of threatened or declining species (L for biodiversity loss) on the total number of species, human population size, GDP PPP per capita or per capita income (A for affluence), A^2^, and the Gini ratio of income inequality. For power models, we regressed log(L) on the logs of the same independent variables as are in the linear models, except that the quadratic affluence term in this case is [log(A)]^2^. We performed Shapiro-Wilk tests for normality of residuals (the “shapiro.test” and “residuals” commands in R) on the linear and power models shown in Table 1. Finally, we applied the “glm.nb” command (in R's MASS library) to the untransformed dependent and independent variables, in order to parameterize and evaluate negative binomial models (also shown in Table 1).

The raw data for these analyses can be found in Tables S2 and S3 of the supplementary information. We re-analyzed these data with statistical outliers removed, having defined the latter as any country or state flagged by R in at least one of the four diagnostic graphs elicited by the “plot” command (residuals vs. fitted values, standardized residuals vs. theoretical quantiles, standardized residuals vs. fitted values, or standardized residuals vs. leverage). The five countries and six states listed in the Results section are all of and only the societies that meet this criterion.

In addition to the models described in Table 1, we tried models with different time lags between socioeconomic variables and biodiversity loss. In other words, we re-did the regressions described above, but with socioeconomic data from different years: for countries, all years from 1975 through 1997; and for US states, 1979/1980, 1989/1900, and 1999/2000 (in addition to 1969/1970). For the sake of comparability with the models focused on in the Results section, we used power models at the country level, and linear models at the state level. See Table S1 in the supplementary information for the results of our analyses of different time lags.

Further analyses involved percent changes in socioeconomic variables over time, rather than values at a single time; controls for geography, as well as transitions away from communism, at the country level; and a measure of biodiversity gain, rather than loss, at the US state level. For countries, we used the changes over time in socioeconomic variables between 1981 and 1995 (two years with a reasonable separation in time for which a relatively high number of Gini ratios were available). For states, the changes over time were between 1969/1970 and 1999/2000 (the full temporal range available from our sources). Our geographical dummy variables are listed in the Results section. We also used a dummy variable to classify countries as ex-communist. Such countries include all that were formerly part of the Soviet bloc, except for Moldova, where a communist government was elected and has remained in power since 2001. Our measure of biodiversity gain in US states is the number of permanent resident bird species with significant increases in abundance 1966–2005, as described in the Results.

## Supporting Information

Table S1 Economic inequality in models with different time lags between socioeconomic variables and biodiversity loss. The dependent and independent variables are the same as in Tables 1, S2, and S3; except for the different time lags. Models at the country scale are power models; those at the US state scale are linear. The data used for the analyses reported in Table S1 are available upon request from the authors.
(0.07 MB DOC)

Table S2 Raw data for countries. Sources given in main text.
(0.12 MB DOC)

Table S3 Raw data for US states. Sources given in main text.
(0.12 MB DOC)
