# Supplementary material for: Economic Inequality Predicts Biodiversity Loss
Source: PLoS One. 2007 May 16;2(5):e444. doi: 10.1371/journal.pone.0000444 (PMC1864998; doi:10.1371/journal.pone.0000444)
Supplement: Table S1 — Economic inequality in models with different time lags between socioeconomic variables and biodiversity loss. The dependent and independent variables are the same as in Tables 1, S2, and S3; except for the different time lags. Models at the country scale are power models; those at the US state scale are linear. The data used for the analyses reported in Table S1 are available upon request from the authors. (0.07 MB DOC) [file pone.0000444.s001.doc]

| *Countries* |  |  |  |
| --- | --- | --- | --- |
| **Year of socioeconomic data** | **Sample size** | **Parameter estimate for the Gini ratio of household income inequality** | ***P* value of the Gini ratio of household income inequality** |
| 1975 | 23 | 0.29 | 0.71 |
| 1976 | 21 | 0.17 | 0.81 |
| 1977 | 22 | 0.54 | 0.50 |
| 1978 | 20 | 0.45 | 0.62 |
| 1979 | 23 | 1.32 | 0.22 |
| 1980 | 26 | 1.14 | 0.06 |
| 1981 | 32 | 1.39 | 0.01 |
| 1982 | 23 | 0.86 | 0.15 |
| 1983 | 21 | 1.37 | 0.06 |
| 1984 | 25 | 0.46 | 0.34 |
| 1985 | 25 | 0.87 | 0.15 |
| 1986 | 33 | 0.77 | 0.06 |
| 1987 | 28 | 0.65 | 0.30 |
| 1988 | 37 | 1.61 | 7.8 x 10^-4^ |
| 1989 | 45 | 1.76 | 6.4 x 10^-6^ |
| 1990 | 45 | 1.22 | 4.3 x 10^-3^ |
| 1991 | 44 | 0.98 | 0.01 |
| 1992 | 52 | 0.96 | 0.03 |
| 1993 | 43 | -0.03 | 0.96 |
| 1994 | 42 | 0.52 | 0.16 |
| 1995 | 46 | 0.43 | 0.37 |
| 1996 | 41 | 0.79 | 0.14 |
| 1997 | 33 | 1.51 | 0.048 |
| *US states* |  |  |  |
| **Year of socioeconomic data** | **Sample size** | **Parameter estimate for the Gini ratio of family income inequality** | ***P* value of the Gini ratio of family income inequality** |
| 1969/1970 | 45 | 33.6 | 0.02 |
| 1979/1980 | 45 | 39.2 | 0.02 |
| 1989/1990 | 45 | 26.9 | 0.07 |
| 1999/2000 | 45 | 14.2 | 0.36 |
