# Supplementary material for: Economic Inequality Predicts Biodiversity Loss
Source: PLoS One. 2007 May 16;2(5):e444. doi: 10.1371/journal.pone.0000444 (PMC1864998; doi:10.1371/journal.pone.0000444)
Supplement: Table S2 — Raw data for countries. Sources given in main text. (0.12 MB DOC) [file pone.0000444.s002.doc]

| **Country** | **Plant and vertebrate species 2004** | **Human population 1989** | **GDP PPP per capita 1989** (current int. $) | **Gini ratio of household income inequality 1989** | **Threatened plant and vertebrate species 2004** |
| --- | --- | --- | --- | --- | --- |
| Argentina | 11,387 | 32,124,000 | $7,666 | 0.480 | 176 |
| Australia | 19,463 | 16,638,000 | $16,215 | 0.373 | 338 |
| Austria | 3,698 | 7,682,000 | $17,501 | 0.260 | 23 |
| Bangladesh | 5,952 | 101,710,000 | $912 | 0.338 | 85 |
| Brazil | 60,322 | 146,858,000 | $5,446 | 0.615 | 663 |
| Bulgaria | 4,196 | 8,789,000 | $5,608 | 0.226 | 35 |
| Chile | 6,222 | 12,948,000 | $4,589 | 0.524 | 123 |
| China | 35,082 | 1,139,265,000 | $1,229 | 0.360 | 769 |
| Costa Rica | 13,864 | 2,999,000 | $5,152 | 0.460 | 222 |
| Denmark | 2,051 | 5,130,000 | $18,616 | 0.332 | 24 |
| Dominican Republic | 6,250 | 6,967,000 | $3,535 | 0.515 | 102 |
| Estonia | 2,018 | 1,586,000 | $7,533 | 0.276 | 8 |
| Finland | 1,653 | 4,968,000 | $17,642 | 0.302 | 15 |
| Georgia | 4,841 | 5,469,000 | $4,142 | 0.284 | 33 |
| Ghana | 5,000 | 15,054,000 | $1,238 | 0.417 | 160 |
| Guatemala | 10,134 | 8,693,000 | $2,627 | 0.590 | 200 |
| Honduras | 7,119 | 4,723,000 | $1,977 | 0.565 | 204 |
| Hungary | 2,751 | 10,396,000 | $9,463 | 0.269 | 26 |
| India | 26,769 | 832,409,000 | $1,287 | 0.354 | 529 |
| Italy | 6,477 | 56,659,000 | $16,755 | 0.358 | 56 |
| Jamaica | 3,914 | 2,354,000 | $2,790 | 0.485 | 262 |
| Japan | 7,491 | 123,076,000 | $17,663 | 0.376 | 160 |
| Kyrgyzstan | 4,803 | 4,333,000 | $1,851 | 0.260 | 13 |
| Latvia | 1,604 | 2,710,000 | $8,072 | 0.259 | 15 |
| Malaysia | 17,539 | 17,384,000 | $4,156 | 0.484 | 873 |
| Mexico | 29,510 | 82,644,000 | $5,838 | 0.520 | 707 |
| Moldova | 2,080 | 4,347,000 | $3,009 | 0.254 | 22 |
| Netherlands | 1,857 | 14,852,000 | $17,305 | 0.321 | 27 |
| New Zealand | 3,094 | 3,371,000 | $13,005 | 0.366 | 135 |
| Norway | 2,321 | 4,221,000 | $19,778 | 0.367 | 24 |
| Panama | 11,759 | 2,362,000 | $3,421 | 0.567 | 308 |
| Russian Federation | 12,667 | 147,695,000 | $8,237 | 0.265 | 121 |
| Sierra Leone | 3,127 | 4,009,000 | $706 | 0.679 | 82 |
| Singapore | 3,003 | 2,945,000 | $10,944 | 0.390 | 84 |
| Slovakia | 3,620 | 5,234,000 | $8,878 | 0.221 | 29 |
| Spain | 5,983 | 39,154,000 | $13,214 | 0.309 | 90 |
| Sweden | 2,390 | 8,506,000 | $17,319 | 0.304 | 23 |
| Tajikistan | 5,491 | 5,171,000 | $1,870 | 0.276 | 22 |
| Thailand | 13,648 | 53,854,000 | $3,264 | 0.493 | 220 |
| Uganda | 6,541 | 17,134,000 | $671 | 0.422 | 115 |
| Ukraine | 5,678 | 51,766,000 | $7,191 | 0.265 | 41 |
| United Kingdom | 2,738 | 56,595,000 | $17,030 | 0.386 | 45 |
| United States | 22,575 | 252,934,000 | $22,069 | 0.421 | 582 |
| Uruguay | 3,003 | 3,085,000 | $5,704 | 0.413 | 49 |
| Venezuela | 23,675 | 19,250,000 | $4,500 | 0.440 | 218 |
