# Supplementary material for: Economic Inequality Predicts Biodiversity Loss
Source: PLoS One. 2007 May 16;2(5):e444. doi: 10.1371/journal.pone.0000444 (PMC1864998; doi:10.1371/journal.pone.0000444)
Supplement: Table S3 — Raw data for US states. Sources given in main text. (0.12 MB DOC) [file pone.0000444.s003.doc]

| **State** | **Permanent resident bird species 2005** | **Human population 1970** | **Income per capita 1969** (1999 US dollars) | **Gini ratio of family income inequality 1969** | **Declining permanent resident bird species 1966-2005** |
| --- | --- | --- | --- | --- | --- |
| Alabama | 20 | 3,444,165 | $9,026 | 0.393 | 6 |
| Arizona | 32 | 1,770,900 | $11,442 | 0.363 | 3 |
| Arkansas | 15 | 1,923,295 | $8,345 | 0.404 | 3 |
| California | 47 | 19,953,134 | $14,079 | 0.357 | 7 |
| Colorado | 22 | 2,207,259 | $12,100 | 0.349 | 5 |
| Connecticut | 11 | 3,031,709 | $15,135 | 0.336 | 1 |
| Florida | 19 | 6,789,443 | $11,913 | 0.398 | 6 |
| Georgia | 20 | 4,589,575 | $10,285 | 0.381 | 4 |
| Idaho | 14 | 712,567 | $10,300 | 0.350 | 2 |
| Illinois | 19 | 11,113,976 | $13,615 | 0.342 | 3 |
| Indiana | 16 | 5,193,669 | $11,960 | 0.322 | 4 |
| Kansas | 18 | 2,246,578 | $11,410 | 0.362 | 2 |
| Kentucky | 16 | 3,218,706 | $9,447 | 0.392 | 2 |
| Louisiana | 17 | 3,641,306 | $9,077 | 0.403 | 3 |
| Maine | 14 | 992,048 | $9,926 | 0.328 | 1 |
| Maryland | 18 | 3,922,399 | $13,628 | 0.349 | 6 |
| Massachusetts | 12 | 5,689,170 | $13,276 | 0.334 | 2 |
| Michigan | 15 | 8,875,083 | $13,078 | 0.329 | 4 |
| Minnesota | 18 | 3,804,971 | $11,835 | 0.346 | 5 |
| Mississippi | 16 | 2,216,912 | $7,499 | 0.427 | 2 |
| Missouri | 18 | 4,676,501 | $11,500 | 0.369 | 4 |
| Montana | 16 | 694,409 | $10,503 | 0.349 | 2 |
| Nebraska | 14 | 1,483,493 | $10,896 | 0.355 | 4 |
| Nevada | 5 | 488,738 | $13,845 | 0.332 | 1 |
| New Hampshire | 12 | 737,681 | $11,629 | 0.317 | 1 |
| New Jersey | 14 | 7,168,164 | $14,313 | 0.341 | 5 |
| New Mexico | 24 | 1,016,000 | $9,494 | 0.389 | 5 |
| New York | 18 | 18,236,967 | $14,056 | 0.369 | 4 |
| North Carolina | 18 | 5,082,059 | $9,638 | 0.372 | 2 |
| North Dakota | 11 | 617,761 | $9,618 | 0.369 | 2 |
| Ohio | 17 | 10,652,017 | $12,462 | 0.331 | 4 |
| Oklahoma | 18 | 2,559,229 | $10,495 | 0.387 | 4 |
| Oregon | 29 | 2,091,385 | $12,264 | 0.345 | 3 |
| Pennsylvania | 20 | 11,793,909 | $11,944 | 0.334 | 5 |
| South Carolina | 15 | 2,590,516 | $8,972 | 0.375 | 6 |
| South Dakota | 12 | 665,507 | $9,299 | 0.386 | 1 |
| Tennessee | 17 | 3,923,687 | $9,599 | 0.390 | 4 |
| Texas | 42 | 11,196,730 | $10,877 | 0.380 | 10 |
| Utah | 17 | 1,059,273 | $10,507 | 0.330 | 2 |
| Vermont | 10 | 444,330 | $10,799 | 0.341 | 0 |
| Virginia | 18 | 4,648,494 | $11,671 | 0.379 | 5 |
| Washington | 21 | 3,409,169 | $13,078 | 0.335 | 0 |
| West Virginia | 16 | 1,744,237 | $9,089 | 0.371 | 2 |
| Wisconsin | 17 | 4,417,731 | $11,812 | 0.326 | 1 |
| Wyoming | 14 | 332,416 | $11,278 | 0.340 | 1 |
